# Latent HIV-Exosomes Induce Mitochondrial Hyperfusion Due to Loss of Phosphorylated Dynamin-Related Protein 1 in Brain Endothelium

**DOI:** 10.1007/s12035-021-02319-8

**Published:** 2021-02-14

**Authors:** Partha K. Chandra, Ibolya Rutkai, Hogyoung Kim, Stephen E. Braun, Asim B. Abdel-Mageed, Debasis Mondal, David W. Busija

**Affiliations:** 1 Department of Pharmacology, Tulane University School of Medicine, 1430 Tulane Avenue, SL-83, New Orleans, LA 70112 USA; 2 Department of Urology, Tulane University School of Medicine, New Orleans, LA 70112 USA; 3 Tulane University National Primate Research Center, Covington, LA 70433 USA; 4 Department of Microbiology, Debusk College of Osteopathic Medicine, Knoxville, TN 37932 USA

**Keywords:** Cerebral microvascular endothelium, Exosomes, HIV-1, Mitochondrial hyperfusion

## Abstract

Damage to the cerebral vascular endothelium is a critical initiating event in the development of HIV-1-associated neurocognitive disorders. To study the role of mitochondria in cerebral endothelial dysfunction, we investigated how exosomes, isolated from both cell lines with integrated provirus and HIV-1 infected primary cells (HIV-exosomes), accelerate the dysfunction of primary human brain microvascular endothelial cells (HBMVECs) by inducing mitochondrial hyperfusion, and reducing the expression of phosphorylated endothelial nitric oxide synthase (p-eNOS). The quantitative analysis of the extracellular vesicles (EVs) indicates that the isolated EVs were predominantly exosomes. It was further supported by the detection of exosomal markers, and the absence of large EV-related protein in the isolated EVs. The exosomes were readily taken up by primary HBMVECs. HIV-exosomes induce cellular and mitochondrial superoxide production but reduce mitochondrial membrane potential in HBMVECs. HIV-exosomes increase mitochondrial hyperfusion, possibly due to loss of phosphorylated dynamin-related protein 1 (p-DRP1). HIV-exosomes, containing the HIV-Tat protein, and viral Tat protein reduce the expression of p-DRP1 and p-eNOS, and accelerate brain endothelial dysfunction. Finally, exosomes isolated from HIV-1 infected primary human peripheral blood mononuclear cells (hPBMCs) produce more exosomes than uninfected controls and reduce both p-DRP1 and p-eNOS expressions in primary HBMVECs. Our novel findings reveal the significant role of HIV-exosomes on dysregulation of mitochondrial function, which induces adverse changes in the function of the brain microvascular endothelium.

**Supplementary Information:**

The online version contains supplementary material available at 10.1007/s12035-021-02319-8.

## Introduction

Thirty years after the beginning of the acquired immune deficiency syndrome (AIDS) epidemic, the introduction of combined antiretroviral therapy (cART) has moved the management of human immunodeficiency virus-1 (HIV-1) infection toward the suppression of viral load. Unfortunately, there has been a parallel growth in treatment-related complications. Nearly 30−60% of HIV-1 infected patients suffer from HIV-associated neurocognitive disorders (HAND), despite the suppression of HIV replication by cART to nearly undetectable viral levels in most patients [1–4]. Neuropathological complications in AIDS patients have been associated with abnormalities in the cerebral endothelium and the blood-brain barrier (BBB) [5, 6]. The mechanisms, by which HIV-1 infection causes these pathological conditions in brain endothelium, are not well understood. Several studies have shown that dysfunction/injury of HBMVECs results from inflammatory cytokines secreted in response to immune cell activation by HIV-1 [7–9] and/or by the effects of secreted viral-proteins, particularly HIV-1 envelope protein (env) gp120 [10, 11], Tat [12–14], and the non-structural Nef protein [14]. Recent literature indicates that a large proportion of extracellular vesicles (EVs) released by HIV-1 infected cells contain HIV-1 Env [15], gp120 [16], HIV-1 Gag [16], the Nef protein [17–20], trans-activation response elements [21, 22], and EV-associated cytokines [23]. Since their discovery, EVs have been shown to be an important means of communication between cell types [24]. A few studies have portrayed the role of EVs in neuropathogenesis. EV trafficking may have either neuroprotective or neurotoxic effects, depending on the origin and composition of vesicles. Previous studies have shown that compared with healthy controls, HIV-infected individuals have more abundant plasma exosomes [25, 26]. Dalvi et al. [27] reported that activated monocytic EVs increase inflammatory responses in brain endothelial cells. Raymond et al. [28] reported that EVs-containing Nef released by HIV-1 infected microglia, disrupted BBB integrity, and increased BBB permeability. However, little is known about the effect of exosomes released by replication defective, latent, HIV-1 infected cells on primary HBMVECs.

The physiology and pathophysiology of endothelial cells are intimately associated with the functional status of mitochondria [29]. Recently, functional connections between mitochondria and lysosomes have been described [30]. Defects in one of these organelles can induce damage in the other, signaling the presence of a mitochondrial-lysosomal axis [31]. A dysfunctional mitochondrial-lysosomal axis in combination with abnormal EV trafficking has recently been implicated in the pathogenesis of neurodegenerative diseases including Alzheimer’s [32], Parkinson’s [33], and Huntington’s [34] diseases. In this connection, Ma et al. [35] reported that HIV Tat protein decreased mitochondrial membrane potential and increased apoptosis of HBMVECs. However, HIV-exosome mediated brain endothelial dysfunction involving impairment of mitochondrial function has not yet been explored.

We hypothesized that exosomes released by latent HIV-infected cells (HIV-exosomes) accelerate brain endothelial dysfunction by mitochondrial dysregulation. We provide evidence that HIV-exosomes induce cellular/mitochondrial superoxide production and decrease mitochondrial membrane potential. We show that HIV-exosomes induce mitochondrial hyperfusion due to loss of p-DRP1 and the reduced expression of p-eNOS in HBMVECs. Our findings provide a previously unknown HIV-exosome mediated mitochondrial-dependent mechanism for brain endothelial dysfunction that offers new therapeutic directions for the treatment of HIV-associated neuropathogenesis.

## Materials and Methods

### Cell Culture

Primary human brain microvascular endothelial cells (HBMCECs; catalog #ACBRI376) culture media and reagents were purchased from Cell Systems, Kirkland, WA, USA. Cells were maintained in complete classic medium with serum and culture boost (4Z0-500) and Bac-Off (4Z0-644). Passage reagent group (4Z0-800) was used for cell propagation according to the suggested protocol. Briefly, cells were washed with passage reagent group-1 (PRG-1), detached with PRG-2, and the enzymatic reaction stopped with ice-cold PRG-3. Cells were kept on ice and centrifuged at 200×g for 7 min at 4°C. After draining all supernatant except ~ 100 μL, the pellet was loosened by flicking the tube several times. One or two drops of culture boost were added to the pellet, and then, the cells were resuspended in the complete classic medium. Cells were seeded 1:3 or 1:5 in T75 flasks coated with attachment factor (4Z0-210) and incubated at 37°C with 5% CO_2_ in 95% relative humidity. Cells were fed with fresh media every 48 h and were used between passage 6 and 9. Latent HIV-1infected T-cells, J-Lat(9.2) (Cat#9848), and pro-monocytes (U1, Cat#165) were obtained from AIDS Reagent Program, Division of AIDS, NIAID, NIH, Bethesda, MD, USA. Jurkat and U937, normal parental T cell line of J-Lat(9.2) and promonocytic cell line of U1 respectively, were purchased from American Type Culture Collection (VA, USA). TZM-blue cells, a luciferase (Luc) reporter line, were used to measure viral infectivity. These cell lines were cultured in RPMI-1640 media supplemented with 10% fetal bovine serum (FBS), 1% penicillin–streptomycin, and 2 mM l-glutamine (Sigma-Aldrich, St. Louis, MO, USA). Cells were passaged twice a week at a ratio of 1:5.

### Antibodies and Chemicals

Various antibodies were purchased from the following suppliers: HIV-1 Tat (#ab43014) from Abcam, Cambridge, MA, USA; against phospho-DRP1 (#3455), Alix (#2171), GRP94 (#2104), and p-eNOS (#9571) from Cell Signaling Technology, Danvers MA, USA; against CD81 (#sc-23962), CD63 (#sc-5275), and TSG101 (#sc-7964) from Santa Cruz Biotechnology, Dallas, TX, USA; β-actin (#A5441) from Sigma-Aldrich; total DRP-1 (#611112), total eNOS (#610296) from BD Transduction Laboratory, San Jose, CA, USA. Vybrant DiD cell-labeling dye for exosome staining was purchased from Molecular Probes (Eugene, OR, USA). Dihydroethidium (DHE) (Thermo Fisher Scientific, Waltham, MA, USA) was used to detect cytosolic superoxide and MitoSOX Red (Molecular Probes) for the detection of mitochondrial superoxide. To label mitochondria, MitoTracker Red and MitoTracker Green were used (Molecular Probes). Phorbol 12-myristate 13-acetate (PMA), PX866, Dynasore, and Cytochalasin D were from Cayman Chemical (Ann Arbor, MI), Ionomycin was from Sigma-Aldrich, and recombinant human IL-2 (rIL-2) was from Miltenyi Biotec (Auburn, CA, USA). GW4896 was from Selleckchem (Houston, TX).

### Isolation of Exosomes

Equal numbers of latent HIV-1 infected or uninfected cells were cultured in exosome-free culture media. Seventy-two hour post-culture, conditioned media (CM) was collected and stored at -80°C until needed. The cell survival profile at the time of EV isolation was determined by MTT assay. Exosomes from the CM of latent HIV-1 infected or uninfected cells were isolated by a modified differential ultracentrifugation protocol, as previously reported (Datta et al. [36]). Briefly, the CM was centrifuged at 300×g for 10 min and then centrifuged again at 20,000×g for 30 min to remove larger microvesicles. The resulting supernatants were transferred to fresh tubes and filtered through a 0.8-μm membrane filter (EMD Millipore, Burlington, MA, USA). The filtered samples were centrifuged for 2 h at 100,000×g to pellet the enriched exosomes (Avanti^TM^ J30I, Beckman Coulter, Brea, CA, USA). Pellets were then re-suspended in PBS and centrifuged at 100,000×g for another hour. Lastly, the exosome pellets were re-suspended in ~ 500–1000 μL of PBS, aliquoted, and stored at -80°C until used.

### Characterization of Exosomes by the qNano-IZON System

Isolated exosomes were characterized by qNano-IZON (Izon, Cambridge, MA, USA) by measuring the concentration (particles/mL), size-distribution, and particle diameter. The tunable resistive pulse sensing technology (qNano-IZON system) allows exosome detection by driving vesicles through a pore using a combination of electrophoretic and convective flow induced by applied voltage and external pressure across the pore, respectively (Datta et al. [37]). Initially, we calibrated the voltage, stretch, pressure, and baseline current using two standard beads: CPC100 (mode diameter, 115 nm [Izon] and a concentration of 1.1 × 10^13^ particles/mL) and CPC70 (mode diameter of 70 nm [Izon] and a concentration of 1.9 × 10^13^ particles/mL). Finally, we optimized the system at a stretch of 47.99 mm, with a voltage of 0.58 V and a pressure level of 5.0 mbar. For analyses, 40 μL of diluted sample was placed in the upper fluid cell under identical conditions. The upper fluid cell was washed with PBS to remove residual particles after each application to prevent cross-contamination. NP100 pore type is applicable to the intended measurement for 50–200 nm size range of the extracellular vesicles. For analysis with NP100, the samples were filtered by 0.45-μm Millex-HV Syringe Filter (EMD Millipore). The data analysis was performed with the qNano-IZON software.

### Exosome Labeling

Vybrant DiD cell-labeling dye (Molecular Probes) was used for exosome labeling. Initially, 300 μg of freshly isolated exosomes was resuspended in 1 mL of PBS, and then, 5 μL of the DiD-dye was added according to the manufacturer’s instructions. Exosomes with dye were incubated for 15 min at 37 °C; then, 1 mL of 1% BSA in PBS was added, mixed, and kept for 2 min at 37 °C and centrifuged at 32,500 rpm (100,000×g) for 70 min at 4 °C (Optima^TM^ TLX Ultracentrifuge, Beckman Coulter). The DiD-tagged exosome pellet was re-suspended in 80 μL of PBS. The concentration of exosomes was measured by Nanodrop (Thermo Fisher Scientific), aliquoted with a small volume of 20 μL, and kept at -80°C until used.

### Exosome Uptake Assay

An exosome uptake assay was performed using a method described by Datta et al. [36] and modified for these experiments. Exosome uptake by primary HBMVECs was measured in a time- and dose-dependent manner. Initially, 1 × 10^5^ HBMVECs (passage # 7) were cultured in a coated six-well plate. The following day, concentrations of DiD-labeled exosomes (1 μg/mL or 10 μg/mL) isolated from latent HIV-1 infected cells [U1 Exo and J-Lat(9.2) Exo] or uninfected control cells (U937 Exo and Jurkat Exo) were exposed to HBMVEs. Cells were harvested at different time points and the number of DiD-positive cells were detected by the MACSQuant Analyzer 10 system (Miltenyi Biotech Inc., San Diego, CA, USA). The MACSQuant Analyzer 10 system is configured with forward and side scatter channels off the 635 nm laser and includes fluorescent channels with detection channels based on excitations from 405 nm, 488 nm, and 635 nm lasers. To optimize EV detection, a side scatter trigger was set to reduce electronic noise with a 0.22 μm Millex-HV Syringe Filter (EMD Millipore) using the filtered non-EV containing control samples (PBS). A fluorescent trigger was also used on the R1 channel, which has 635 nm excitation and a bandpass filter allowing 635/730 nm wavelengths of emitted light to further reduce electronic noise. Nano fluorescent standard particles (Cat. No. NFPPS-52-4 K, Spherotech Inc. Lake Forest, IL, USA) with Vybrant DiD cell-labeling dye were used as a reference standard to calibrate the experiment. The MACSQuant Analyzer 10 syringe driven fluidics system allows for the volumetric samples measurement, which can be quantified on an event/μL or event/mL basis.

### Isolation of Exosomes from Uninfected and HIV-1 Infected hPBMCs

Human peripheral blood mononuclear cells (hPBMCs) were isolated using discontinuous density-gradient centrifugation with lymphocyte-separation media (Thermo Fisher Scientific). Equal numbers (0.5 × 10^6^ cells) of hPBMCs were cultured in the presence of 10 ng/mL of phorbol 12-myristate 13-acetate (PMA) with 1 μg/mL of ionomycin for 24 h in a T-25 flask with 6 mL RPMI-1640 media. Next day, the activated cells were resuspended in 3 mL of fresh RPMI-1640 media, and the infected cells were resuspended with 300 μL of HIV-1_Bal_ (HIV-1 p24 = 307 ng/mL) for 6 h. Another 2 mL of fresh media was added, and cells were kept for 24 h at 37 °C. Next day, the infected cells were removed, washed twice with PBS, and cultured the uninfected and HIV-1 infected hPBMCs with 6 mL exosome-free RPMI media with 5 μL of human rIL-2 (stock concentration 100 IU/μL). After 6 days of infection, exosomes were isolated from uninfected and HIV-1 infected culture supernatant using QIAGEN exoEasy Maxi kit (Germantown, MD, USA), according to the manufacturer’s instruction. Interestingly, we observed an undetectable amount of HIV-1 p24 in the exosomes isolated from uninfected hPBMCs, whereas more than 2000 pico-gram/mL of HIV-1 p24 was detected in exosomes isolated from HIV-1-infected hPBMCs.

### HIV-1 Infectivity Assay

The Luciferase reporter based TZM-bl cell line was used to measure HIV-1 infectivity (Kim et al. [38]) after exposure with either HIV-positive U1 Exo/J-Lat(9.2) Exo or HIV-negative U937 Exo/Jurkat Exo. Briefly, TZM-bl cells (5 × 10^4^ cells/well) were cultured in a 48-well plate one day before the experiment. Cells were exposed to 50 μg/mL of exosomes for 24, 48, and 72 h, washed with PBS, lysed with 100 μL of 1× lysis buffer, centrifuged (5000 rpm for 5 min), and then underwent firefly luciferase measurement using a luciferase assay system from Promega (Madison, WI, USA). Cells exposed to cell free virus (CFV) (HIV-IIIB and HIV-Bal) were used as positive controls. The relative light units for both control and exosomes or CFV-exposed cells were determined by using a Lumat LB 9507 Ultra-Sensitive Luminometer (Berthold Technologies, Waltham, MA).

### Detection of HIV-1 p24 by ELISA

HIV-1 p24 protein levels in exosomes were determined by an enzyme-linked immunosorbent assay (ELISA) kit from ABL Inc. (Rockville, MD) as described previously [39]. Briefly, ELISA plates were washed with wash buffer and disruption buffer was applied. Exosomes were then added to the designated wells, covered with plate-sealer tape, and incubated at 37°C for 1 h. Wells were then washed with wash buffer, conjugate solution was added, and were incubated again at 37°C for 1 h. Following another washing round, peroxidase substrate was added to each well and incubated at 37°C for 30 min. Afterward, stop solution was added and the absorbance of each well (at 450 nm) was quantified using a BioTek Synergy^HTX^ plate reader (BioTek Instruments, Winooski, Vermont, USA). The HIV-1 p24 levels (pg/mL) were calculated using the standard curves.

### Detection of Superoxide Production

We used DHE and MitoSOX Red to measure cytosolic and mitochondrial superoxide production, respectively, in primary HBMVECs. These probes are oxidized to form intermediate probe-derived radicals that are successively oxidized to generate the corresponding fluorescent products [40]. Initially, 1 × 10^4^ HBMVECs (passage # 8) were cultured in a coated 12-well plate. After 48 h, cells were treated with 25 μg/mL of HIV-positive U1 Exo/J-Lat(9.2) Exo or HIV-negative U937 Exo/Jurkat Exo. After 24 h, cells were stained with either DHE (0.5 μM) or MitoSOX Red (5 μM) and counter stained for nucleus with Hoechst 33342 (Thermo Fisher Scientific) for 30 min. Dyes were then removed, cells were washed, and pictures were taken under fluorescence microscopy.

### Analysis of Mitochondrial Membrane Potential (∆Ψm)

Mitochondrial membrane depolarization was measured by JC-1 dye **(**Molecular Probes) in primary HBMVECs after exposure to HIV-positive U1 Exo/J-Lat(9.2) Exo or HIV-negative U937 Exo/Jurkat Exo. Initially, 1 × 10^4^ HBMVECs (passage # 8) were cultured in a coated 12-well plate. After 48 h, cells were treated with 25 μg/mL of exosomes. After 24 h, cells were stained with 10 μg/mL JC-1 dye for 20 min at 37 °C. Nucleus in the cells was counter stained with Hoechst 33342 for 30 min, dyes were removed, cells were washed, and pictures were taken under fluorescence microscopy.

### Western Blotting

Our standard laboratory protocol, described previously, was followed to prepare the cell lysates and to perform the immunoblots [41]. In brief, cells were lysed in phosphatase and protease inhibitors containing ice-cold NP40 lysis buffer (Invitrogen, Frederick, MD, USA). The supernatant was used for Pierce BCA protein assay (Thermo Scientific) and the proteins were separated using a 4–20% SDS-PAGE gradient gel and transferred onto a PVDF membrane. To block the non-specific binding sites, casein blocking buffer (Li-Cor, Lincoln, NE, USA) was used. The same buffer used to dilute primary antibodies. The membranes were washed with Tris-buffered saline containing 0.1% Tween-20 (Sigma-Aldrich). After an overnight incubation with primary antibodies at 4 °C, membranes were washed and incubated again with respective secondary antibodies, either goat anti-rabbit IgG at 1:2500 dilution (#7074S, Cell Signaling Technology) or goat anti-mouse IgG at 1:5000 dilution (#7076P2, Cell Signaling Technology) at room temperature for 1 h. Chemiluminescence (LumiGLO, Gaithersburg, MD, USA) and autoradiography were used to visualize the final reaction. In some cases, immunoblot signals were captured using the ImageQuant Las 300 (GE Healthcare, Piscataway, NJ, USA) system. Densitometry of the immune-bands was performed using the ImageJ software (NIH, Bethesda, MD, USA, http://imagej.nih.gov/ij/).

### Statistical Analysis

All data were summarized using descriptive statistics and reported as means and standard deviations, where meaningful results were presented graphically. Data comparison was performed using the unpaired Student’s *t* test and one-way ANOVA with Tukey’s post hoc analysis. *p* < 0.05 was considered statistically significant.

## Results

### Secreted EVs Were Mostly Exosomes

**Fig. 1:**
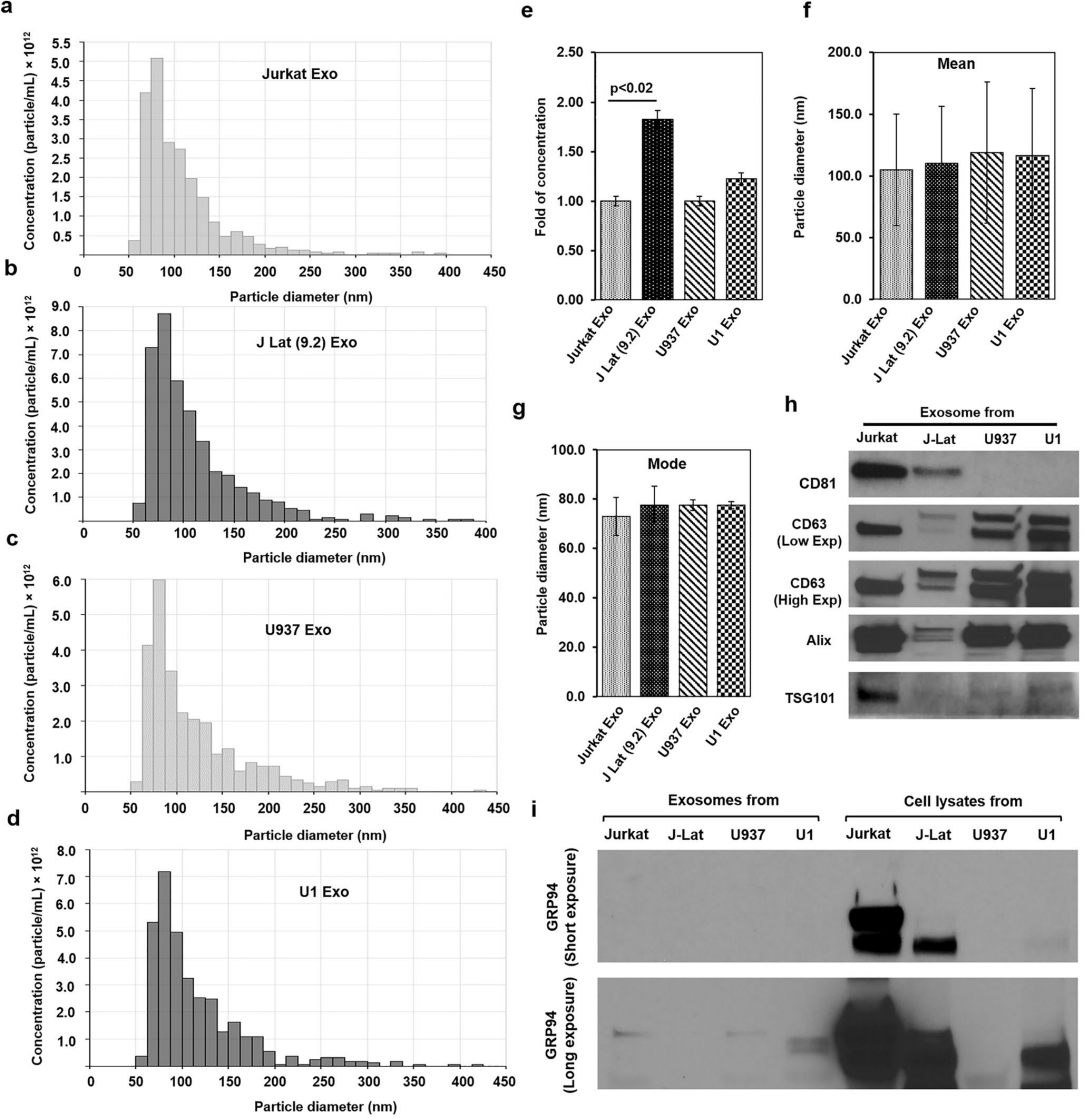
Characterization of exosomes isolated from uninfected and latent HIV-1 infected T-cells and promonocytes. Extracellular vesicles (EVs) were isolated from **a** HIV(−) Jurkat T-cells (Jurkat Exo), **b** latent HIV(+) J-Lat (9.2) T-cells [J Lat (9.2) Exo], **c** HIV(−) promonocytes (U937 Exo), and **d** latent HIV(+) promonocytes (U1 Exo) and the distribution of concentrations (particles/mL) with the particle diameter (nm) were measured by qNano-IZON system. **e** The concentration of exosomes isolated from HIV(−) and latent HIV(+) cells were compared (fold change). Simultaneously, the mean (**f**) and mode (**g**) of the isolated exosomes diameters were also measured. **h** Exosomes isolated from different cells were lysed, quantified, and the exosomal markers were detected by western blots from equal amounts (20 μg) of lysates. **i** GRP94, exclusively present in large EVs, was detected in the cell lysates but was not prominent in the different exosomes. Significant changes (*p* < 0.05) are presented as *p* values

EVs were isolated by ultracentrifugation technique from latent HIV-1 infected J-Lat(9.2) and U1 cells and their uninfected Jurkat and U937 cells. The cell survival profile at the time of EV isolation was presented in Supplementary Fig. 1. The qNano-IZON quantitative analysis showed that the average concentration of EVs (combined exosomes and microvesicles) in the CM of HIV(+) J-Lat(9.2) and U1 cells (3.95 × 10^13^ and 3.33 × 10^13^ particles/mL, respectively) was higher than HIV(−) Jurkat and U927 cells (2.35 × 10^13^ and 2.88 × 10^13^ particles/mL, respectively) (Fig. 1a–d). Quantitative analysis with the NP-100 nanopore showed that the EV concentration isolated from J-Lat(9.2) cells [J-Lat(9.2) Exo] was significantly higher (*p* < 0.02) than Jurkat cells (Jurkat Exo) (Fig. 1e). The mean particle diameters of Jurkat Exo (105 ± 45 nm), J-Lat(9.2) Exo (110 ± 47 nm), U937 Exo (119 ± 57 nm), and U1 Exo (116 ± 55 nm) were comparable (Fig. 1f). Similarly, the mode of the particle diameters of Jurkat Exo (73 ± 8 nm), J-Lat(9.2) Exo (77 ± 8 nm), U937 Exo (77 ± 2 nm), and U1 Exo (77 ± 1 nm) was also similar (Fig. 1g). Western blot analysis showed the enrichment of exosomal protein markers (CD81, CD63, Alix, and TSG101) in the isolated EVs (Fig. 1h). On the other hand, GRP94, a marker for large EVs, demonstrated a large presence in cell lysates but was in very low abundance in the exosome-enriched fraction (Fig. 1i). Therefore, the particle diameter plus the enrichment of exosomal proteins clearly indicated that the isolated EVs were predominantly exosomes.

### Exosomes Isolated from Latent HIV-1 Infected Cells Do Not Support HIV-1 Replication

**Fig. 2:**
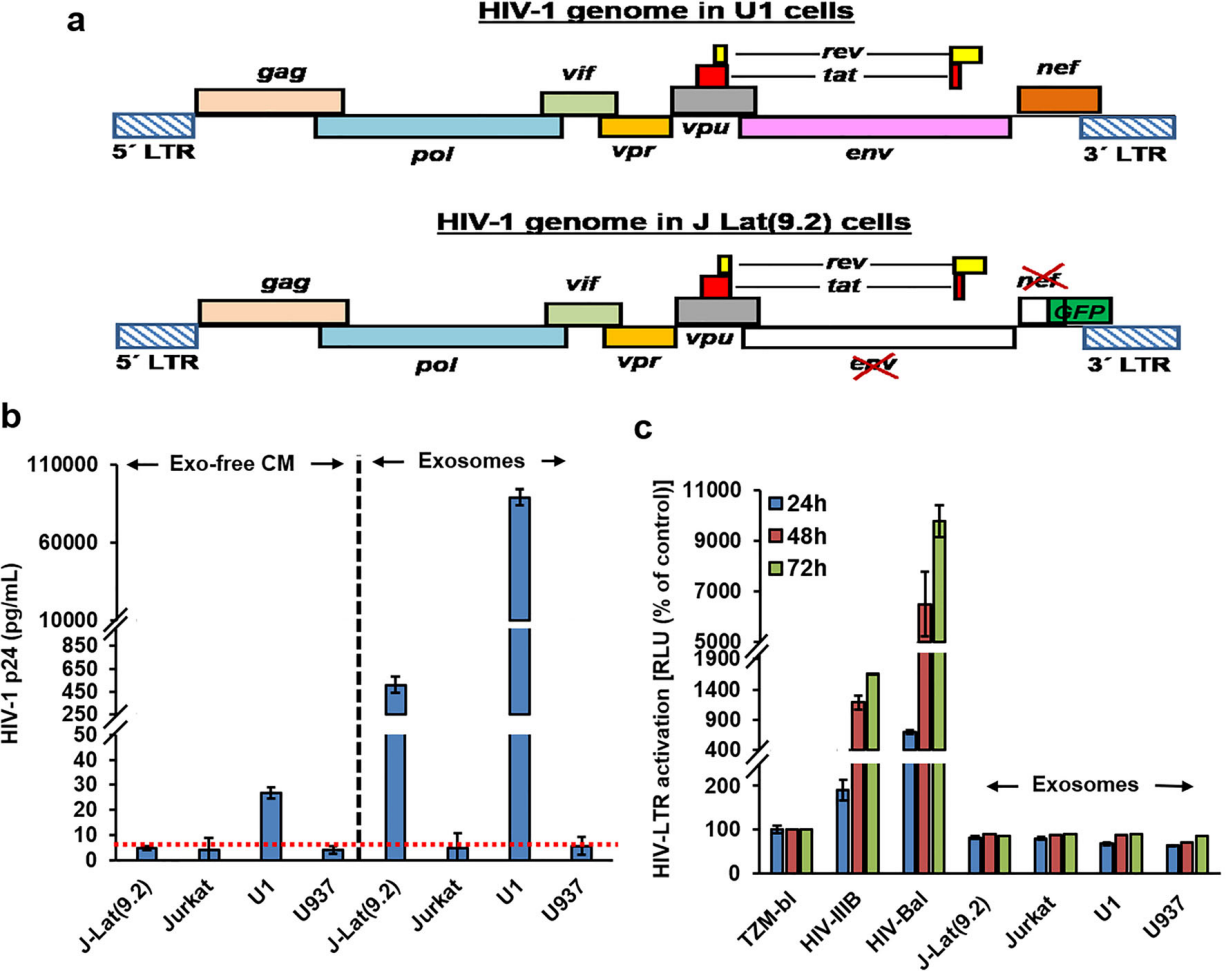
Exosomes from latent HIV-1 infected cells do not support HIV-1 replication. **a** Upper diagram: HIV-1 viral genome present in latent HIV(+) promonocytes (U1 cells). Lower diagram: HIV-1 viral genomes present in latent HIV(+) T-cells [J Lat(9.2) cells]. The coding region of viral env and one of the accessory proteins, Nef, were deleted from the HIV-1 genome present in J Lat(9.2) cells. The GFP reporter was inserted in the deleted region. **b** The presence of the core structural protein, HIV-1 p24, was measured by ELISA in the isolated exosomes and also in the exosome-free, conditioned media (Exo-free CM). The red dotted line indicates the assay detection limit. **c** To study HIV-1 replication, HIV-LTR regulate luciferase reporter based TZM-bl cells were exposed to the exosomes (50 μg/mL) for 3 days. HIV-IIIB (CXCR4-tropic) and HIV-1_Bal_ (CCR5-tropic) viruses were used as positive controls. Cells were lysed and luciferase activity was measured and compared. Error bars show mean ± standard deviation (SD), *n* = 3 independent experiments per group

The genome organization of U1 and J-Lat(9.2) cells are presented in Fig. 2a. All of the HIV-protein coding genes were intact in latently infected U1 cells (Fig. 2a, upper panel) whereas HIV env and one of the accessory proteins, HIV-Nef, coding regions were deleted from the HIV-1 genome integrated in J-Lat(9.2) cells (Fig. 2a, lower panel). Initially, we measured the level of HIV-1 p24 protein in different isolated exosomes and in the corresponding exosome-free conditioned media (Exo-free CM) by ELISA (Fig. 2b). Very low levels of HIV-1 p24 (nearly 30 pg/mL) were detected in Exo-free CM from U1 cells but were below the detection limit (nearly 5 pg/mL) in Exo-free CM from other cells. Alternatively, very high levels of HIV-1 p24 protein were present in U1 Exo (nearly 90,000 pg/mL). HIV-1 p24 protein was also detected in J-Lat(9.2) Exo (nearly 500 pg/mL). As expected, HIV-1 p24 protein was below the detection limit in Jurkat Exo and U937 Exo (Fig. 2b). Finally, we studied the HIV-1 infectivity by using Luc reporter based TZM-bl cells exposed to exosomes (50 μg/mL) isolated from HIV(+)/HIV(−) cells. Both CXCR4-tropic (HIV-IIIB) and CCR5-tropic (HIV-1_BAL_) cell free virus (CFV) were used as positive controls. After 24 h of infection both HIV-IIIB and HIV-1_BAL_ infected cells show time-dependent increase of viral replication, whereas no viral replication was observed when the TZM-bl cells were exposed to J-Lat(9.2) Exo or U1 Exo (Fig. 2c).

### Exosomes Were Readily Taken Up by Primary HBMVECs

**Fig. 3:**
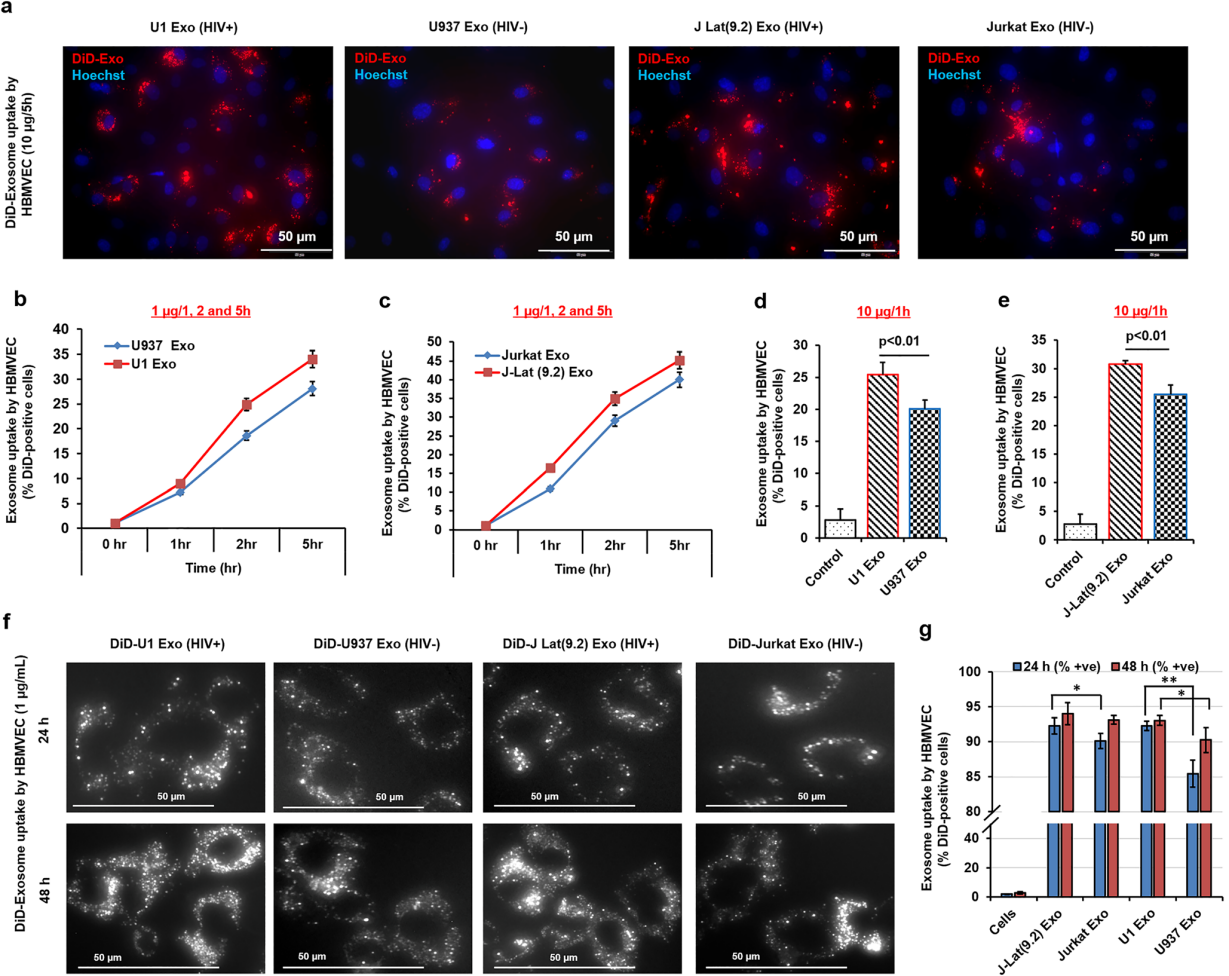
The differential uptake of exosomes isolated from uninfected and latent HIV-1 infected cells by primary HBMVECs. **a** Cells were exposed to DiD-label exosomes (10 μg/mL) for 5 h, washed twice with PBS, and the red fluorescence of DiD-exosomes were monitored under fluorescent microscope. The red dots indicate the uptake of DiD-exosomes by HBMVECs. **b** and **c** Using low concentrations of exosomes (1 μg/mL), the kinetics of the exosome uptake by HBMVECs with time (as indicated) was compared by detecting the number of DiD-positive cells by MACSQuant Analyzer. **d** and **e** To study high-affinity uptake, cells were incubated with 10 μg/mL exosomes for 1 h, and the exosome uptake by HBMVECs was assessed by measuring the number of DiD-positive cells by MACSQuant Analyzer. **f** For maximum uptake, cells were exposed to DiD-exosome (1 μg/mL) for 24 and 48 h, washed, and qualitatively measured by detecting DiD-positive cells under fluorescence microscopy, and (g) quantitatively measured by MACSQuant Analyzer. For better image quality, the brightness and contrast were adjusted uniformly within experiments using either Adobe Photoshop 7.0 or ImageJ software (version 1.50). Error bars show mean ± SD, *n* = 3 independent experiments per group, and significant changes are presented as *p* values (*p < 0.01, **p < 0.001)

DiD-tagged exosome uptake by primary HBMVECs was qualitatively detected by fluorescence microscopy. Uptake of HIV(+) DiD-tagged U1 Exo and J-Lat(9.2) Exo by HBMVECs was much higher than HIV(−) DiD-tagged U937 Exo and Jurkat Exo (Fig. 3a). Quantitative analysis (MACSQuant Analyzer 10 system) showed that the concentration and uptake of exosomes by HBMVECs were increased in a time-dependent manner (Fig. 3b–g). When exposed to a very low concentration of exosomes (1 μg/mL), 10–15% of DiD-positive cells were detected within 1 h, which gradually increased to 45% over 5 h. Interestingly, the tendency of the HIV(+) exosome uptake by HBMVECs was higher than control HIV(−) exosomes by HBMVECs (Fig. 3b–c). Likewise, when cells were exposed to a higher concentration of exosomes (10 μg/mL), higher uptake (20–30%) was also observed within 1 h (Fig. 3d–e), and the uptake of U1 Exo (Fig. 3d) and J-Lat(9.2) Exo (Fig. 3e) was significantly higher (*p* < 0.01) than U937 Exo and Jurkat Exo, respectively. To show maximum uptake by primary HBMVECs, cells were exposed to 1 μg/mL of exosomes for 24 h and 48 h and examined by fluorescence microscopy. Most cells were DiD-positive within 24 h and the fluorescence was stable even 48 h post-exposure (Fig. 3f). Quantitatively, 24 h post-exposure, 92.2% of J-Lat(9.2) Exo were positive with significantly higher (*p* = 0.03) uptake than Jurkat Exo (90%). Uptake of U1 Exo by HBMVECs (92.2%) was also significantly higher (*p* = 0.003) than U937 Exo (85.4%). The exosome uptake was comparable even 48 h post-exposure, except that uptake of U937 Exo by the HBMVECs increased from 85.4% to 90.2% but was significantly less (*p* = 0.04) than U1 Exo (93%) (Fig. 3g). Furthermore, we studied the inhibition of U1 Exo uptake by using different small molecule uptake inhibitors, e.g., dynasore (inhibit endocytosis), cytochalasin D (inhibit actin polymerization), PX866 (inhibit PI3K pathway), and GW4896 (a neutral sphingomyelinase inhibitor and also used as exosome biogenesis inhibitor). Interestingly, we observed that PI3K pathway was very important for exosome uptake by HBMVECs. The U1 Exo uptake was significantly (*p* < 0.05) inhibited by potent PI3K inhibitor, PX866. Dynasore and GW4869 were also significantly (*p* <0.05) inhibited the U1 Exo uptake by HBMVECs (Supplementary Fig. 2).

### Latent HIV-Exosomes Significantly Induced Cellular and Mitochondrial Superoxide Production but Reduced Mitochondrial Membrane Potential (∆Ψ_m_) in Primary HBMVECs

**Fig. 4:**
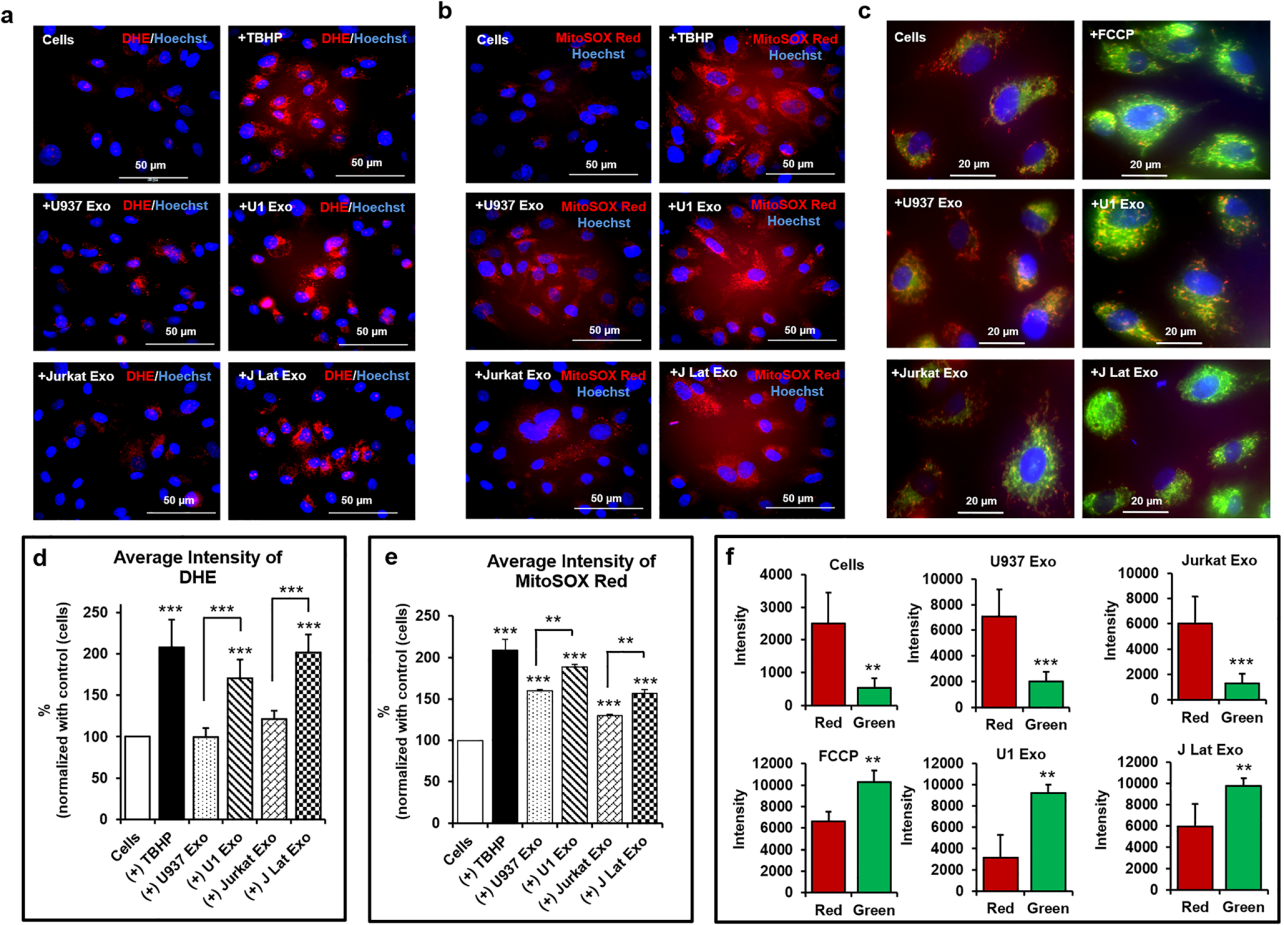
Latent HIV-exosomes induce cellular and mitochondrial superoxide production and reduce mitochondrial membrane potential in primary HBMVECs. **a** The increased production of cytosolic superoxide was detected by DHE after 24 h treatment with 25 μg/mL of exosomes. Cells were stained with 0.5 μM DHE for 30 min, washed twice with PBS, and the pictures were taken under fluorescence microscopy with ×40 magnification. **b** Similarly, to measure the mitochondrial superoxide, cells were exposed to specific exosomes (25 μg/mL) for 24 h. After treatment, cells were stained with 5 μM MitoSOX Red for 30 min, washed twice with PBS, and pictures acquired by fluorescence microscopy with ×40 magnification. Tert-Butyl Hydrogen Peroxide (TBHP/200 μM for 3 h) was used as positive control. **c** The mitochondrial membrane potential was detected by JC-1 dye. Cells were treated with exosomes (25 μg/mL) for 24 h. Treated cells were incubated for 20 min at 37 °C with 10 μg/mL of JC-1 dye. Finally, cells were washed with PBS, and pictures were taken under fluorescence microscopy with ×60 magnification. FCCP was used as positive control. Hoechst 33342 dye was used for nuclear staining. For better image quality, the brightness and contrast were adjusted uniformly within experiments using either the Adobe Photoshop 7.0 or the ImageJ software (version 1.50). The average fluorescence intensity of **d** DHE, **e** MitoSOX Red, and **f** JC-1 dye was quantified by the ImageJ software (version 1.50). Error bars show mean ± SD, *n* = 3 independent experiments per group, and significant changes are presented as *p* values (**p < 0.001, ***p < 0.0001)

Cells exposed to U1 Exo and J-Lat(9.2) Exo showed increased numbers of dihydroethidium (DHE)-positive cells compared with cells exposed to U937 Exo and Jurkat Exo in primary HBMVECs (Fig. 4a). MitoSOX Red measured superoxide production in the mitochondrial matrix (mitoROS) and due to the positively charged cationic triphenylphosphonium subunits, it rapidly targeted the mitochondria. Similar to DHE, cells exposed to U1 Exo and J-Lat(9.2) Exo produced more mitochondrial superoxide than control exosomes (U937 Exo and Jurkat Exo) (Fig. 4b). Cells exposed to U937 Exo produced modest amounts of mitochondrial superoxide compared with other controls (Fig. 4b). The loss of ∆Ψ_m_ (measure by JC-1 dye) by U1 Exo and J-Lat(9.2) Exo was much higher than U937 Exo and Jurkat Exo in primary HBMVECs (Fig. 4c). We used the ImageJ software to measure the fluorescence intensity of DHE (Fig. 4d), MitoSOX Red (Fig. 4e), and JC-1 dye (Fig. 4f). U1 Exo or J-Lat(9.2) Exo exposed a significantly higher number of cells (*p* < 0.001 or *p* < 0.0001) than either U937 Exo or Jurkat Exo.

### HIV-Exosome Induced Mitochondrial Hyperfusion Due to Loss of p-DRP1

**Fig. 5:**
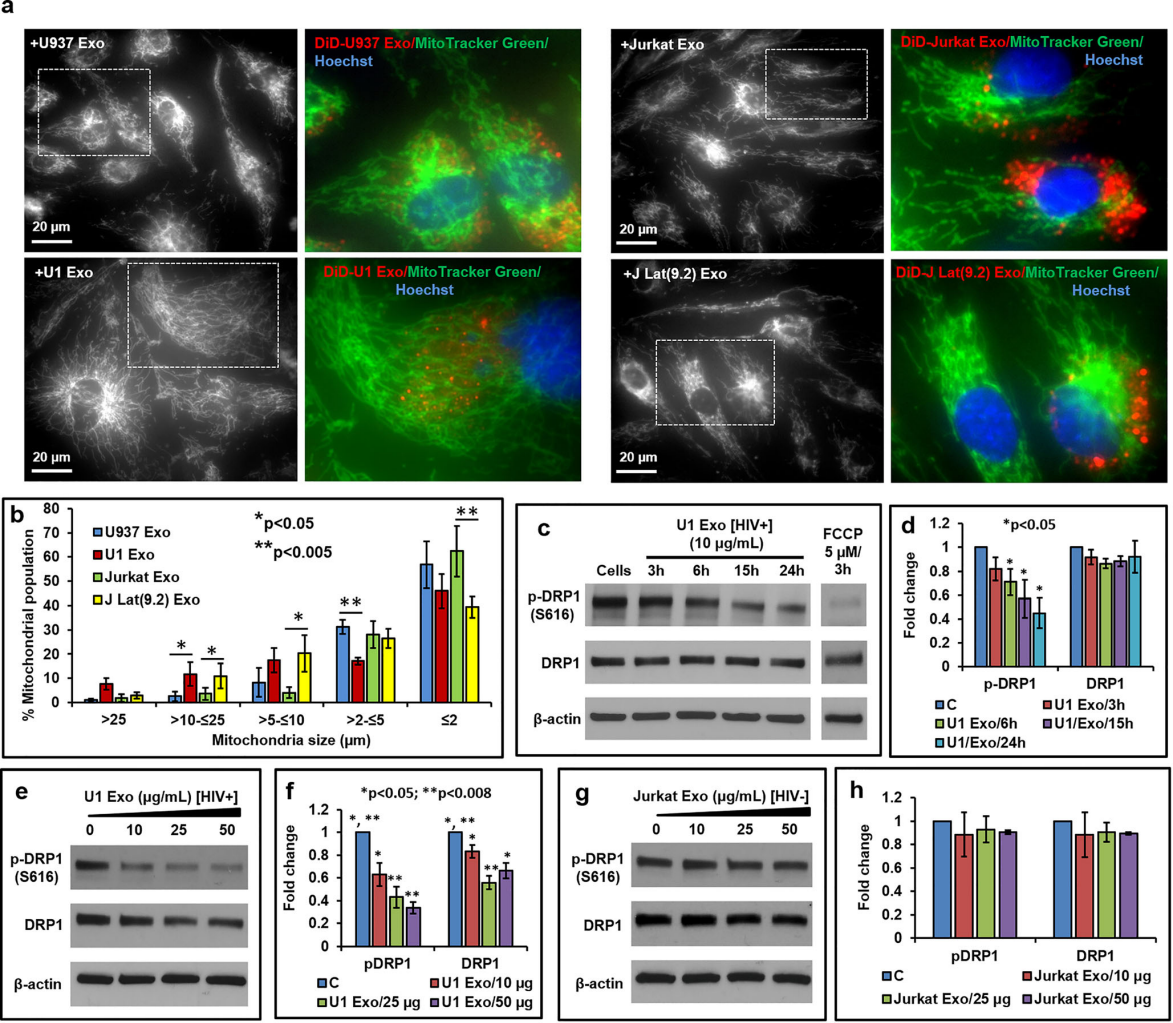
HIV-exosomes induce mitochondrial hyperfusion due to loss of p-Drp-1. **a** The effect of HIV-exosome on the mitochondrial fission and fusion events was studied by a co-localization experiment. After 24 h treatment with DiD-tagged (red) exosomes (25 μg/mL), cells were stained for 1 h with Hoechst 33342 nuclear dye (blue) and 45 min with MitoTracker Green (100 nM). After removal of Hoechst and MitoTracker Green, cells were washed with PBS, and the pictures were taken under fluorescence microscopy with ×60 magnification. For better image quality, the brightness and contrast were adjusted uniformly within experiments using the ImageJ software (version 1.50). **b** The mitochondrial size was measured by the ImageJ/Fiji software. More than 200 mitochondria were counted and compared from each of the given treatment conditions. The mitochondrial count was from at least three cells (*n* = 3) for each treatment condition. Error bars show mean ± SD, and significant changes are presented as *p* values (*p < 0.05, **p < 0.005). Cells were treated in a time-dependent manner as indicated, with representative western blot (**c**), and quantitative analysis (**d**) of p-DRP1 and total DRP1. In the second experiment, cells were treated for 24 h with increasing concentrations of U1 Exo. **e** Representative western blot and **f** quantitative analysis of p-DRP1 and total DRP1. Representative western blot (**g**), and quantitative analysis (**h**) of p-DRP1 and total DRP1 in cells exposed to HIV(−) Jurkat Exo. FCCP was used as positive control. Error bars show mean ± SD, *n* = 3 independent experiments, and significant changes are presented as *p* values

The normal architecture of the mitochondria was observed when primary HBMVECs were exposed to control U937 Exo and Jurkat Exo (Fig. 5a, upper panels), but thread-like, elongated mitochondria was formed when cells were exposed to U1 Exo and J-Lat(9.2) Exo (Fig. 5a, lower panels). Mitochondrial measurement suggested that the percent population of larger size (> 10 to ≤ 25 μm) mitochondria in the cells exposed to U1 Exo and J-Lat(9.2) Exo was significantly (*p* < 0.05) larger than cells exposed to U937 Exo and Jurkat Exo, respectively (Fig. 5b). However, the percent population of smaller size (> 2 to ≤ 5 μm for U1 Exo and ≤ 2 μm for J-Lat(9.2) Exo) mitochondria was significantly (*p* < 0.005) lower than controls (Fig. 5b). To study the effects of exosomes on DRP-1, primary HBMVECs were exposed to either U1 Exo, or Jurkat Exo in a time- and dose-dependent manner. Interestingly, we observed that cells exposed to U1 Exo (10 μg/mL), the p-DRP1 level was significantly (*p* < 0.05) decreased 6 h after exposure and was further decreased at 24 h post-exposure (Fig. 5c–d). Furthermore, both p-DRP1 and total DRP1 levels were significantly (*p* < 0.05) decreased in a dose-dependent manner (10, 25, and 50 μg/mL) after 24 h of exposure to U1 Exo (Fig. 5e–f). Conversely, both p-DRP1 and total DRP1 levels remained unchanged when the cells were exposed to U937 Exo (data not shown) and Jurkat Exo (Fig. 5g–h).

### HIV-Exosomes Contain HIV-1 Tat Protein and Viral Tat Protein Reduce p-DRP1, and p-eNOS Expression in Primary HBMVECs

**Fig. 6:**
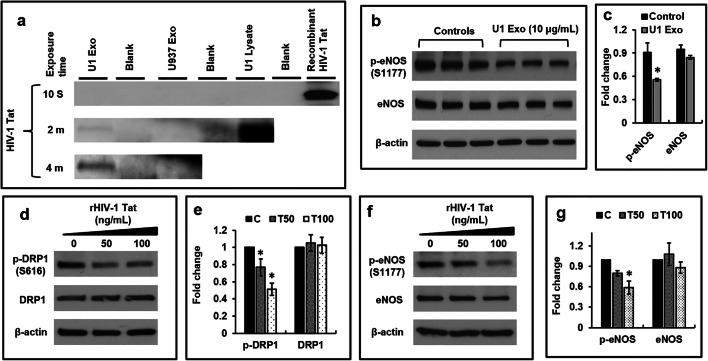
HIV-exosomes contain HIV-1 Tat protein and viral Tat protein reduce p-DRP1, and p-eNOS expression in primary HBMVECs. **a** HIV-1 Tat protein in U1 Exo, U937 Exo, and U1 cell lysate was detected (20 μg protein) by western blotting. Recombinant HIV-Tat protein (50 ng) was used as positive control. **b** Representative western blot of p-eNOS and total eNOS in cells exposed to U1 Exo. **c** Quantitative analysis of p-eNOS and total eNOS. **d** Representative western blot, and **e** quantitative analysis of p-DRP1 and total DRP1 in cells exposed to recombinant HIV-Tat protein. **f** Representative western blot, and **g** quantitative analysis of p-eNOS and total eNOS in cells exposed to recombinant HIV-Tat protein. Error bars show mean ± SD, *n* = 3 independent experiments, and significant changes are presented as *p* values (*p < 0.05)

We detected the HIV-1 Tat protein in U1 Exo and U1 cell lysate but not in HIV(−) U937 Exo by western blotting (Fig. 6a). To address the dysfunction of HBMVECs, we measured the p-eNOS level in cells exposed to HIV-exosome and found that the expression of p-eNOS was significantly (*p* < 0.05) decreased (Fig. 6b–c). Using recombinant HIV-Tat (rHIV-Tat) protein, the expression of p-DRP1 (*p* < 0.05) was decreased in a dose-dependent manner (Fig. 6d–e). Interestingly, we measured the p-eNOS level in cells exposed to rHIV-Tat and found that the expression of p-eNOS was also significantly (*p* < 0.05) decreased (Fig. 6f–g).

### Exosomes Isolated from HIV-1 Infected hPBMCs (HIV+hPBMC-exo) Reduce p-DRP1, and p-eNOS Expression in Primary HBMVECs

**Fig. 7:**
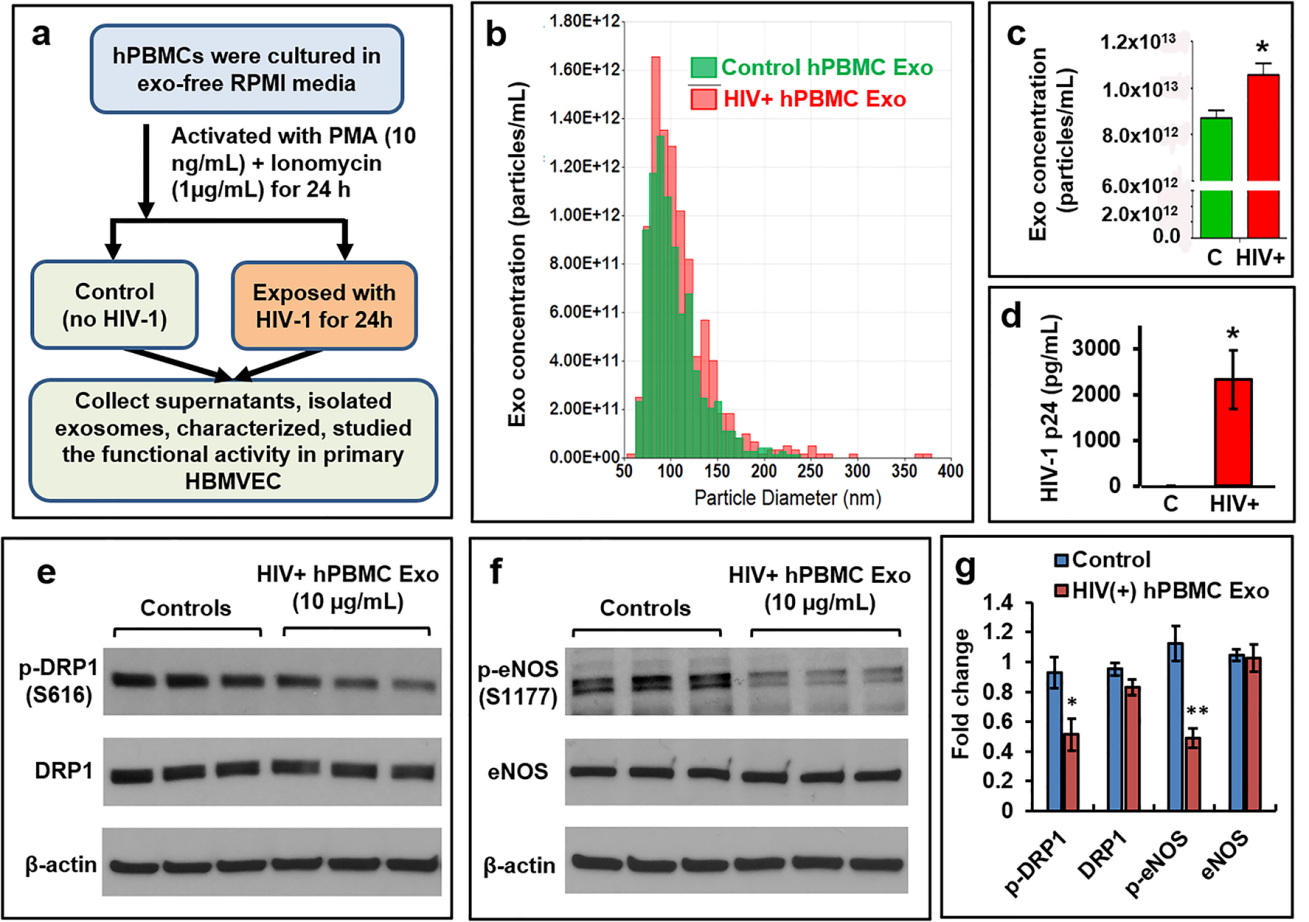
Exosomes isolated from HIV-1 infected hPBMCs reduce the expression of p-DRP1, and p-eNOS in primary HBMVECs. **a** The schematic was indicated the culture, activation, infection, and isolation of exosomes from either HIV-1 infected (HIV+hPBMC Exo) or control hPBMCs. **b** Extracellular vesicles (EVs) were isolated from HIV-1 infected and uninfected hPBMCs, and the distribution of concentrations (particles/mL) with the particle diameter (nm) was measured by qNano-IZON system. **c** The concentration of exosomes isolated from HIV-1 infected and uninfected hPBMCs was compared. **d** The presence of HIV-1 p24 protein in control Exo (C) and HIV+hPBMC Exo was measured by ELISA. Representative western blot of **e** p-DRP1 and total DRP1, and **f** p-eNOS and total eNOS in cells exposed to control Exo and HIV+hPBMC Exo. **g** Quantitative analysis of p-DRP1, total DRP1, p-eNOS, and total eNOS. Error bars show mean ± SD, *n* = 3 independent experiments, and significant changes are presented as *p* values (*p < 0.05, **p < 0.003)

To overcome the limitations of the cell line-based studies, we repeat our key findings in primary HBMVECs that were exposed to HIV+hPBMC Exo by following the steps that were presented in Fig. 7a. The qNano-IZON quantitative analysis was indicated that the size of both control (C) and HIV+hPBMC Exo (HIV+) was almost identical. The isolated EVs were mostly exosomes due to their size of <150 nm (Fig. 7b). Several clinical studies reported that exosome concentration was frequently higher in the blood of HIV+ patients when compared to healthy subjects [25, 26]. Consistent with the clinical reports, we observed that the concentration of HIV+hPBMC Exo was significantly (*p*<0.03) higher that control Exo (Fig. 7c). The presence of HIV-1 p24 protein (measured by ELISA) in HIV+hPBMC Exo indicated that isolated particles were predominantly HIV exosomes (Fig. 7d). Like U1 Exo, HIV+hPBMC Exo also significantly decreased the expression of p-DRP1 (*p*<0.05), and *p*-eNOS (*p*<0.003) in primary HBMVEC (Fig. 7e–g).

## Discussion

**Fig. 8:**
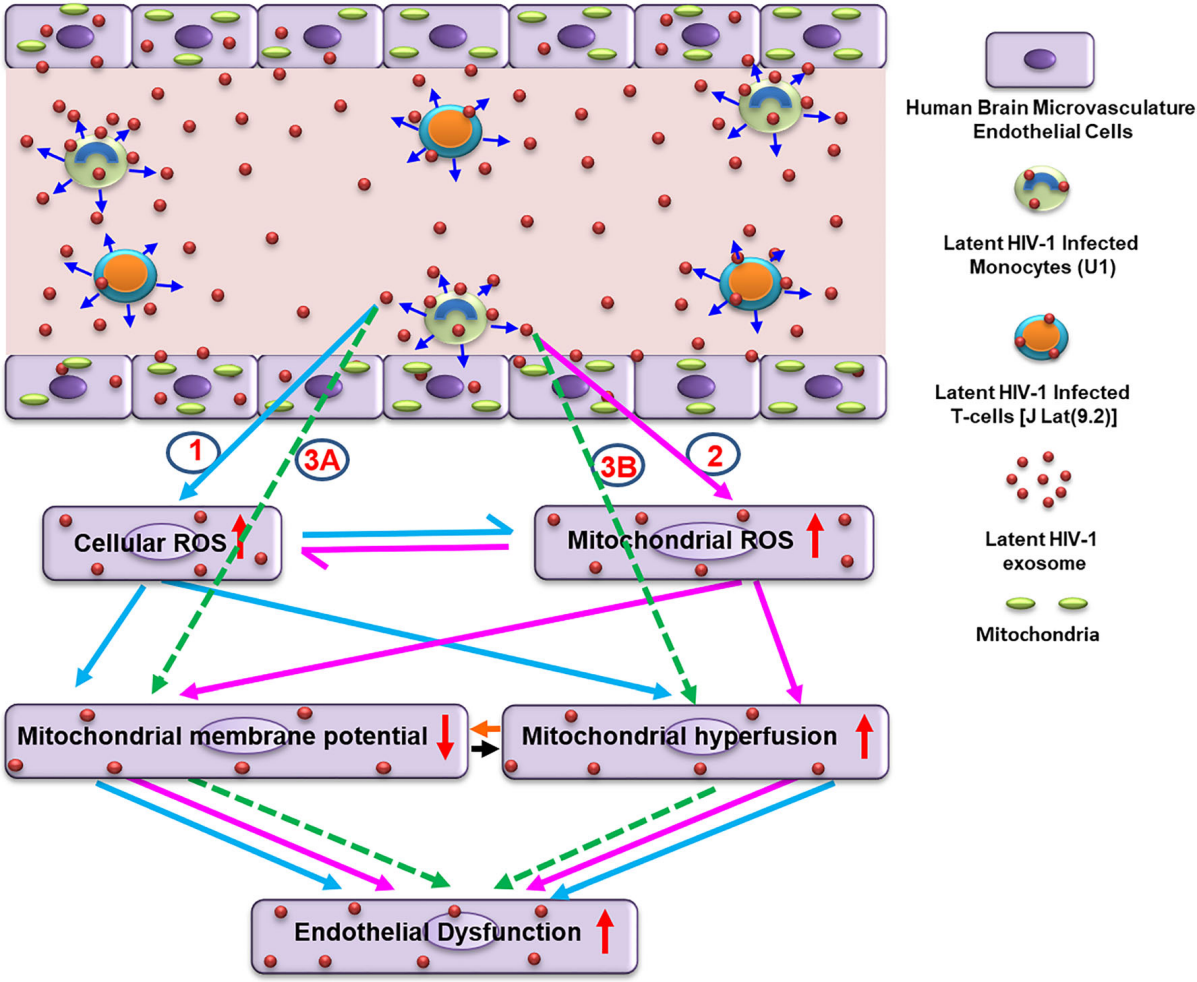
Probable mechanistic pathways by which HIV-exosomes can perturb normal mitochondrial dynamics and cause initial HBMVECs dysfunction leading to brain pathologies. HIV-exosomes are readily taken up by primary HBMVECs and cause dysfunction and damage via multiple pathways involving mitochondria. The following experimental observations were noticed: (1) primary HBMVECs exposed to HIV-exosomes produced cellular ROS that subsequently induced mitochondrial depolarization, and hyperfusion resulting in brain endothelial dysfunction (indicated by light blue arrows). (2) Similarly, HIV-exosomes also generated mitochondrial ROS, which in turn, caused endothelial dysfunction by inducing mitochondrial depolarization, and hyperfusion (indicated by pink arrows). (3) HIV-exosomes could be developed endothelial dysfunction by ROS-independent pathways (indicated by dotted green arrows). Viral/cellular factor(s) could be directly dysregulated the mitochondrial membrane potential (3A), or mitochondrial fission/fusion machinery (3B). Dark blue arrows indicated the release of exosomes by the cells. The black and orange arrows indicated the possible crosstalk within mitochondrial depolarization, and hyperfusion for the development of endothelial dysfunction

HAND is a common neurological complication of HIV/AIDS infection resulting in devastating clinical complications [42, 43]. Damage to the brain endothelium is a critical, initiating event for influx of pathogens, toxins, and immune cells into the brain [44]. Our findings, using HIV-exosomes, clearly indicate a novel role of mitochondria in mediating dysfunction and damage of HBMVECs. We demonstrated that HIV(+) U1 Exo or J Lat(9.2) Exo, which demonstrate replication competence, induced both mitochondrial ROS and mitochondrial depolarization in HBMVECs. We observed that HIV-exosomes or HIV+hPBMCs Exo induced mitochondrial hyperfusion due to the loss of p-DRP1 and the reduced expression of p-eNOS in HBMVECs. To understand the mechanisms, we observed that HIV-exosomes contain HIV-Tat protein that might reduce the expression of p-DRP1 and p-eNOS in HBMVECs. Therefore, HIV-exosomes may perform a crucial role in disrupting the brain microvascular endothelial function to accelerate neuropathogenesis (Fig. 8).

Exosomes are an important means of communication between cell types [24]. Intercellular communication via exosomes can occur by the expression of ligands to receptors on the target cell membrane. Otherwise, internalization of exosomes can occur either by direct fusion with plasma membrane by phagocytosis and micropinocytosis, or by clathrin-mediated endocytosis [45]. Endothelial cells use endolytic pathways rather than fusion mechanisms to uptake exosomes in a time, concentration, and temperature-dependent manner [46]. Consistent with this view, we observed that exosomes were internalized very rapidly by HBMVECs in a dose- and time-dependent manner (Fig. 3a−g). Most experimental evidence suggests that EVs are readily taken up via endocytosis [47–49]. Endocytosis is an umbrella term for a range of molecular internalization pathways [50], including macropinocytosis [49, 51], phagocytosis [47, 49, 52], and clathrin-mediated endocytosis [49]. Phosphatidylinositol-3-kinase (PI3K) is a specific regulator of endocytosis [53]. The signaling factor PI3K activities have been shown to stimulate macropinocytosis [50]. Moreover, PI3Ks also play an important role in phagocytosis [54]. The PI3K inhibitors were used to assess the necessity of functional PI3Ks in EV uptake [52]. Our present study is indicating that PX866, a clinically relevant PI3K inhibitor, significantly inhibits the uptake of U1 Exo in primary HBMVECs (Supplementary Fig. 2). CD46 [55], intercellular adhesion molecule 1, DEC205 [56], as well as various members of the heparan sulfate proteoglycans family [57], are the major receptors for the uptake of exosomes by brain endothelium.

Extracellular vesicles play an important role in HIV infection and pathogenesis. In fact, EVs (especially exosomes) and HIV-1 virions share some crucial aspects regarding their biogenesis, biophysical/molecular properties, and cellular uptake mechanisms [58, 59]. In this study, filtration and ultracentrifugation were used to isolate exosomes by targeting their physical characteristics (mainly density and size), and the exosomes were further characterized. The mean particle diameter (around 110 nm), presence of exosomal proteins (CD81, CD63, Alix, and TSG101), and the low abundance of GRP94, a marker of large EVs [60], indicate that isolated EVs are a mostly exosome-enriched fraction (Fig. 1a–i). This morphological similarity between HIV-1 (110–128 nm) and exosomes (< 150 nm) makes their accurate separation technically challenging [61, 62]. The use of sucrose gradients has had little success [63] because the density of exosomes (1.13–1.19 g/mL) is similar to an HIV-1 virus (1.09–1.16 g/mL) [64]. Moreover, due to their almost identical molecular composition, biochemical separation is also challenging. However, by iodixanol velocity gradient, it might be possible to separate exosomes from HIV-1 particles [63]. In this study, contamination of infectious HIV-1 particles with exosomes can be overlooked because of the use of replication defective latent HIV-1 infected U1 and J-Lat(9.2) cells. The U1 monocytic cell line expresses mutated/inactive Tat [65, 66]. The virus produced by J-Lat(9.2) cells do not express a functional Nef protein and contains a frameshift mutation in the HIV-1 env gene [67] (Fig. 2a). A moderate to a very high level of HIV-1 p24 protein was detected in the exosomal fraction from J-Lat(9.2) and U1 cells, respectively (Fig. 2b) but they failed to show the infectivity as infectious viruses (Fig. 2c). The presence of other viral proteins and RNAs is one of the potential limitations of the present study. However, the results clearly indicate that these isolated exosomes are not contaminated with active or infectious virus particles.

A recent study suggests that activated monocytic EVs increase inflammatory responses in brain endothelial cells [27]. Russell et al. [68] reported that inflammation-derived EVs may alter mitochondrial activity in recipient cells. As a primary regulator of mitochondrial fission, DRP1 is controlled by a wide array of signaling pathways [69]. Altered expression levels and aberrant post-translational modification, including phosphorylation of DRP1, have been linked to various neurodegenerative diseases [70]. Generally, DRP1 deficiency is believed to cause mitochondrial dysfunction due to a failure of a DRP1-dependent mechanism of mitophagy [71]. DRP-1 deficiency has been reported to enhance an accumulation of ROS in human umbilical cord vein endothelial cells [72]. Our present study supports this concept. We observed that the HIV-exosome (U1 Exo) induces ROS (Fig. 4) and increases mitochondrial hyperfusion due to loss of p-DRP1 (Fig. 5a–h). There may be some differences in the reaction between HIV-exosomes and brain endothelium in vitro and in vivo due to use of different passages of cells in different experiments.

To understand the mechanism of HIV-exosome mediated loss of p-DRP1 and brain endothelial dysfunction, we explored the role of HIV-Tat protein in HIV-exosomes (Fig. 6a). We showed that both HIV-exosomes and rHIV-Tat protein reduced the expression of p-DRP1 and p-eNOS in HBMVECs (Fig. 6b–g). HIV-1 Tat has been detected in the sera of HIV patients [73] and recent literature indicates that EVs released by HIV-1 infected cells or HIV patients contain HIV-Tat protein [74, 75]. Rahimian and He [76] showed that HIV-Tat protein is released in the form of exosomes and is biologically active. A recent study has shown that HIV-Tat is persistently expressed in the CSF and CNS of individuals virologically controlled on cART [77]. It has also been reported that HIV-Tat induces production of endothelial ROS [78] and decreases the mitochondrial membrane potential of brain endothelium [35]. Simultaneously, HIV-Tat has been shown to downregulate the expression of eNOS, resulting in reduced NO production and endothelial dysfunction [79, 80]. However, we are the first to explore the possible role of HIV-exosome containing HIV-Tat in brain endothelial dysfunction via impairment of mitochondrial function.

In summary, exosomes are important mediators of intercellular communication and modulate a multitude of signaling pathways in recipient cells via trafficking of cytosolic components in the progress of numerous diseases. However, HIV-exosomes are detrimental to many cell types including HBMVECs via maladaptive mitochondrial fission/fusion and likely lead to brain pathologies. Therefore, targeting exosome-mediating physiological and pathological communications between cells will have significant therapeutic potential in a wide array of diseases.

## Supplementary Information

ESM 1 (JPG 109 kb)

ESM 2 (JPG 251 kb)

## Data Availability

The datasets generated during and/or analyzed during the current study are available from the corresponding author on reasonable request.
