# Supplementary figures and images for: Latent HIV-Exosomes Induce Mitochondrial Hyperfusion Due to Loss of Phosphorylated Dynamin-Related Protein 1 in Brain Endothelium
Source: Mol Neurobiol. 2021 Feb 14;58(6):2974–89. doi: 10.1007/s12035-021-02319-8 (PMC8128843; doi:10.1007/s12035-021-02319-8)

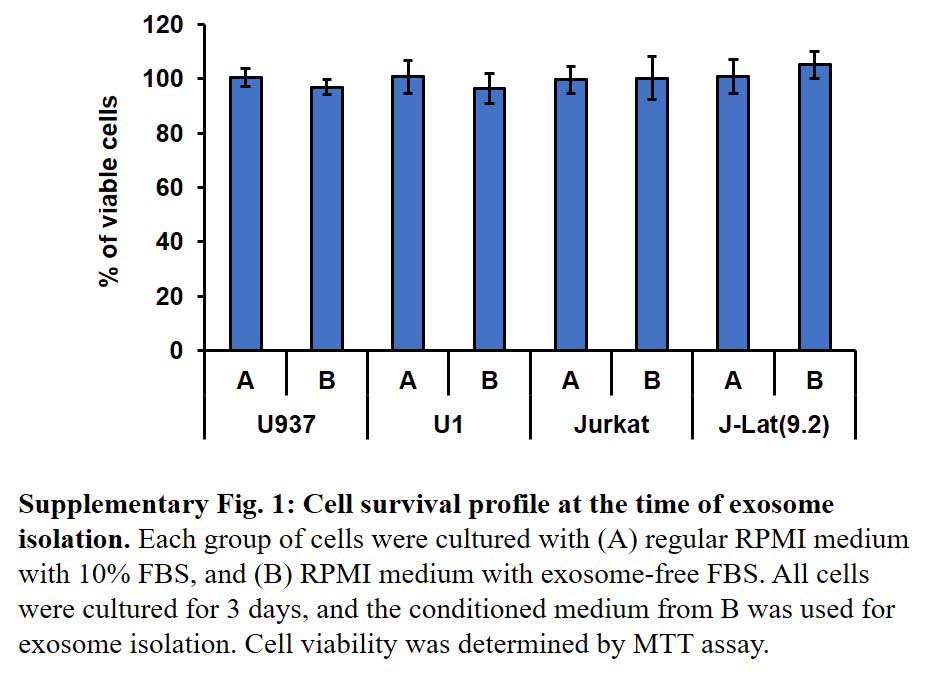

Supplement: Supplementary file 1 — (JPG 109 kb) [file 12035_2021_2319_MOESM1_ESM.jpg]

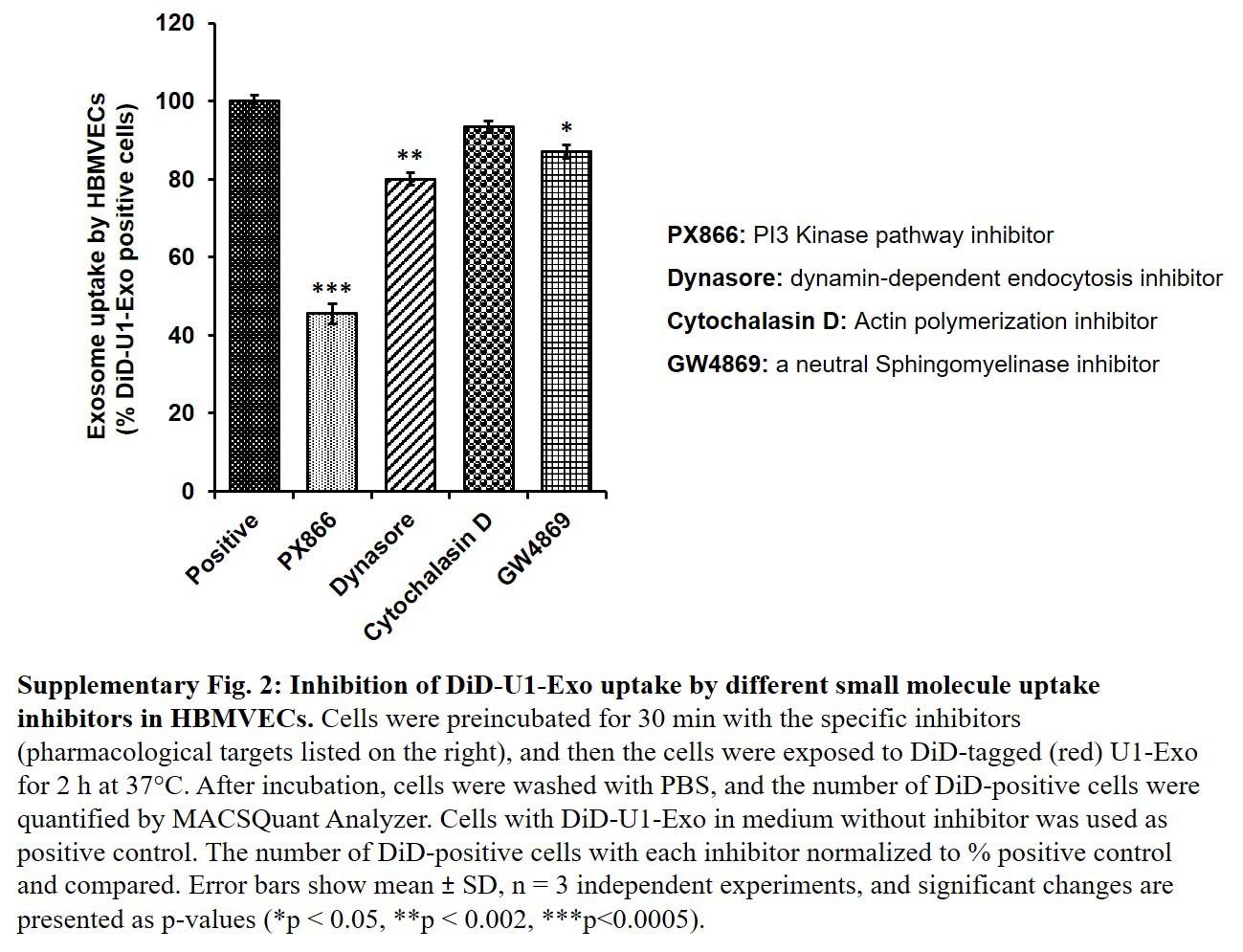

Supplement: Supplementary file 2 — (JPG 251 kb) [file 12035_2021_2319_MOESM2_ESM.jpg]
